# TRANSLATION OF A MEDICARE CLAIMS-BASED FRAILTY ALGORITHM FROM ICD-9-CM TO ICD-10-CM

**DOI:** 10.1093/geroni/igac059.1302

**Published:** 2022-12-20

**Authors:** Keturah Faurot, Emilie Duchesneau, Shahar Shmuel, Jihye Park, Til Stürmer, Michele L Jonsson-Funk, Jennifer L Lund

**Affiliations:** University of North Carolina at Chapel Hill, Chapel Hill, North Carolina, United States; University of North Carolina at Chapel Hill, Chapel Hill, North Carolina, United States; Gillings School of Global Public Health, Chapel Hill, North Carolina, United States; University of North Carolina at Chapel Hill, Chapel Hill, North Carolina, United States; Gillings School of Global Public Health, Chapel Hill, North Carolina, United States; University of North Carolina at Chapel Hill, Chapel Hill, North Carolina, United States; University of North Carolina at Chapel Hill, Chapel Hill, North Carolina, United States

## Abstract

Clinical trials often have insufficient power to estimate drug effects in older adult populations. Medicare claims data are a valuable resource for studying healthcare delivery for older adults. The Faurot frailty index (FFI) is a validated algorithm that uses demographic, enrollment, and ICD-9-CM-based claims information to predict ADL dependency as a proxy for frailty. The original FFI consists of 20 constructs, including those related to geriatric syndromes, of which 16 are based on ICD-9-CM diagnosis codes (e.g., arthritis, dementia). In October 2015, the US healthcare system transitioned to ICD-10-CM. In this study, we updated and validated the FFI for the ICD-10-CM era . We used General Equivalence Mapping (GEM) to translate the ICD-9-CM codes to ICD-10-CM. For each construct, we manually reviewed the ICD-10-CM codes after GEM, assessed the suitability, and plotted the monthly prevalence before and after the transition (2013-2017). Code lists for all but 1 construct required editing after translation using GEM (adding and/or removing codes). We observed increasing monthly prevalence for several constructs, although trend lines were consistent across the ICD-9-CM and ICD-10-CM eras: bladder dysfunction (1.8-2.5%), weakness (2.7-3.9%), and psychiatric illnesses (6.9-9.1%). Rehabilitation services did not translate well using diagnosis codes, however, adding CPT codes improved capture. The updated FFI was strongly associated with frailty-related geriatric outcomes (1-year mortality, hospitalization, SNF admission). Studies of drug effects in older adults should use validated indices, such as the FFI, to reduce bias. Thorough evaluation of claims-based algorithms is essential to support healthcare services research using these measures.

